# Primary Radical Effectiveness: Do the Different Chemical Reactivities of Hydroxyl and Chlorine Radicals Matter for Tropospheric Oxidation?

**DOI:** 10.1021/acsestair.3c00108

**Published:** 2024-05-23

**Authors:** Peter M. Edwards, Cora J. Young

**Affiliations:** † Wolfson Atmospheric Chemistry Laboratories, Department of Chemistry, University of York, York YO10 5DQ, United Kingdom; ‡ National Centre for Atmospheric Science, University of York, York YO10 5DQ, United Kingdom; § Department of Chemistry, York University, Toronto, ON M3J1P3, Canada

**Keywords:** Primary radical, secondary radical, chlorine atom, hydroxyl radical, organic oxidation, box model

## Abstract

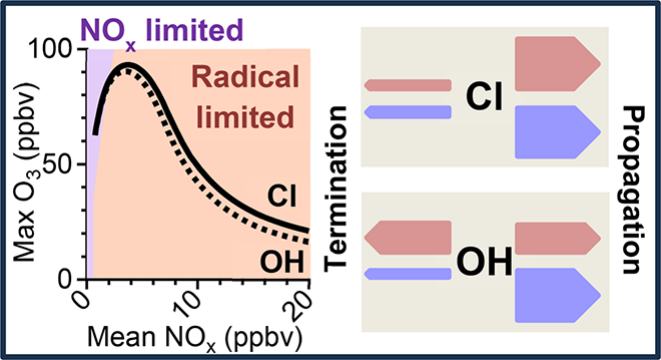

The atmospheric oxidation of organics occurs primarily via reaction cycles involving gas phase radical species, catalysed by nitric oxide (NO), which result in the production of secondary pollutants such as ozone. For these oxidation cycles to occur, they must be initialized by a primary radical, i.e., a radical formed from non-radical precursors. Once formed, these primary radicals can result in the oxidation of organic compounds to produce peroxy radicals that, providing sufficient NO is present, can re-generate “secondary” radicals which can go on to oxidize further organics. Thus, one primary radical can result in the catalytic oxidation of multiple organics. Although the photolysis of ozone in the presence of water vapor to form two hydroxyl (OH) radicals is accepted as the dominant tropospheric primary radical source, multiple other primary radical sources exist and can dominate in certain environments. The chemical reactivity of different radicals to organic and inorganic compounds can be very different, however, and how these differences in radical chemistry impact atmospheric organic oxidation under different atmospheric conditions has not been previously demonstrated. In this work, we use a series of model simulations to investigate the impact of the chemical reactivity of the primary radical on the effectiveness in initializing organic oxidation and thus the production of the secondary pollutant ozone. We compare the chemistries of the OH and atomic chlorine (Cl) radicals and their effectiveness at initializing organic oxidation under different nitrogen oxide and organic concentrations. The OH radical is the dominant tropospheric radical, with both primary and secondary sources. In contrast, Cl has primary sources that show significant spatial heterogeneity throughout the troposphere but is not typically regenerated in catalytic cycles. Both primary OH and Cl can initiate organic oxidation, but this work shows that the relative effectiveness with which they oxidize organics and produce ozone depends on their balance of propagation vs termination reactions which is in turn determined by the chemical environment in which they are produced. In particular, our work shows that in high NOx radical-limited environments, like those found in many urban areas, Cl will be more efficient at oxidizing organics than OH.

## Abstract 2

A box model was used to elucidate the different impacts on tropospheric organic oxidation resulting from reactions initiated with Cl and OH.

## Introduction

**Figure 1:**
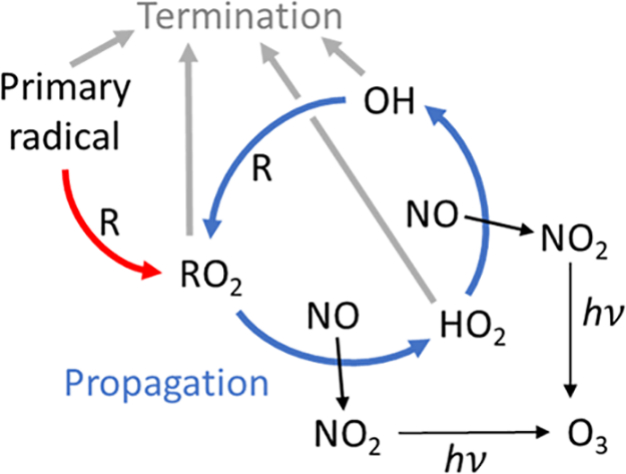
Simplified schematic depicting key reactions in the catalytic oxidation of organics (R) and photochemical production of ozone. Note that termination could be through radical–radical reactions or NO_x_ reactions.

The chemistry of the troposphere is that of oxidation, with highly reactive radical species responsible for the chemical oxidation and eventual removal of emitted trace gases and production of secondary pollutants, such as ozone and secondary particulate matter.^1^ The dominant gas phase tropospheric radical species is the hydroxyl radical (OH)^2^ which initiates oxidative cycles that, in the presence of nitrogen oxides (NO_x_ ≡ NO + NO_2_), result in the catalytic oxidation of organics and the photochemical production of ozone via the reactions shown in Figure 1.

As OH radicals are regenerated during these oxidation cycles a distinction can be made between the primary radical that initiates the cycle shown in Figure 1, for example OH formed through the photolysis of ozone, and secondary radicals which are reformed through radical propagation reactions (i.e. radical reactions where radicals are formed as products and the radical is thus preserved) during the oxidation cycle. The efficiency with which a primary radical can oxidize organics and generate secondary pollutants is thus determined by the number of secondary radicals generated during the subsequent radical propagation reactions before a radical termination reaction occurs. The concept of radical chain length is often used to quantify this efficiency and is defined as the rate of radical propagation reactions divided by the rate of radical termination.^3^ A chain length of >1indicates more radicals propagate than terminate and a chain length of 0 indicates all radicals are lost to termination reactions, and thus the higher the chain length the higher the efficiency of organic oxidation. This is a useful concept when thinking about overall radical chemistry efficiency, however, in the case where we want to compare the effectiveness of different primary radicals in initiating hydrocarbon oxidation it can be more useful to consider the fraction of total radical reactions that are reactions with organic molecules (i.e., organic reaction fraction), and thus initiating the oxidation cycle shown in Figure 1. A radical organic reaction fraction of 1 indicating all of the primary radicals of interest are reacting with organics, and an organic reaction fraction of 0 indicating all of the primary radical of interest are reacting via termination reactions with no secondary radicals formed. As NO_x_ acts as both a catalyst for the cycle shown in Figure 1 and an important radical termination mechanism, via HNO_3_ formation, this chemistry is highly nonlinear with respect to NO_x_ concentration. When considering ozone formation, a secondary pollutant with detrimental health and climate impacts, the nonlinear nature of this chemistry is often classified into either NO_x_ limited or volatile organic compound (VOC)/radical limited regimes.^4^ In the NO_x_ limited case, NO_x_ concentrations are sufficiently low that radical termination occurs predominantly via radical–radical reactions, with increases in NO_x_ concentration acting to increase the radical propagation, and thus overall radical chain length. Once the NO_x_ concentration increases sufficiently for NO_x_ to become the dominant radical termination mechanism, the system transitions into a radical sensitive regime where further increases in NO_x_ concentration act to reduce the radical chain length as the organic reaction fraction decreases as more radicals are lost to termination reactions. Understanding the sensitivities of the chemical regime in a particular environment is thus critical in designing effective policy interventions to tackle secondary pollutants formed via gas phase organic oxidation such as ozone.

Although OH is the dominant tropospheric radical globally, other radicals exist and can play significant roles in certain environments. Chlorine atoms (Cl) are the least understood of the major tropospheric oxidants, with current estimates of their role ranging from highly important^5^ to negligible.^6^ The highly reactive Cl atom is a powerful oxidant of both organic and inorganic compounds, often reacting orders of magnitude faster with organics than the OH radical. This high chemical reactivity means that even at low concentrations, Cl atoms can represent a significant loss for emitted organics.^7^ The production of tropospheric primary Cl atoms is via heterogeneous mechanisms that liberate gas phase Cl atom reservoirs, such as nitryl chloride (ClNO_2_), from particulate chloride (pCl^–^) and their subsequent photolysis or reaction to release a Cl atom.^8^ Primary Cl atoms can react with organics, either via hydrogen abstraction or addition to a carbon–carbon double bond, to produce an organic radical (R.) species. Unlike the analogous OH reaction scheme, however, Cl is generally not regenerated during the oxidation cycles, with the secondary radicals formed being the same as those generated in the OH case. There is the potential for increased Cl recycling from the chlorinated organics produced from Cl addition to alkenes, but the fate of these species is poorly understood. This means that for a Cl organic reaction fraction of >0, organic oxidation initiated by Cl atoms proceeds both via reaction with the primary Cl and secondary OH radicals. This makes methods to assess the true role of Cl oxidation using observations such as hydrocarbon ratios difficult to interpret, as the secondary OH generated from Cl oxidation masks primary Cl reactions in most cases.^9,10^

Chlorine atoms typically react faster than OH radicals, though this is not universally true.^11^ Most organics commonly present in the atmosphere are much more reactive with Cl atoms, where reactivities (i.e., the pseudo-first order loss rates, see SI) for Cl with most aliphatic organics are several times to orders of magnitude higher than those of OH. Among important atmospheric inorganic species, reactivity differences between Cl and OH are smaller, with the exception of O_3_, where Cl reactivity is more than one hundred times higher than OH. In one study where Cl and OH total reactivities in an urban atmosphere were assessed, Cl reactivity was more than ten times higher, with a larger fraction lost to organic reaction.^9^ These differences will affect the impacts of these radicals on tropospheric chemistry, yet these have not been well constrained due to a lack of explicit mechanisms to represent hydrocarbon oxidation by Cl in models.^12−14^ Models that include Cl atom sources typically show an increase in O_3_, which is generally attributed to an increase in the overall number of radicals and thus organic oxidation (e.g.,^15^). While a few radical budgets have incorporated Cl alongside other tropospheric radicals, (e.g.,^16,17^) the budgets do not distinguish between the potential different impacts of Cl and OH caused by their different chemistry. Elucidating the impacts of Cl and OH on tropospheric chemistry remains a challenge because of limited representation of Cl chemistry in model reaction mechanisms.

In this work we use idealized box-model simulations to provide a more complete understanding of the nature of the primary radical (OH vs Cl) on organic oxidation and the production of the secondary pollutant ozone. We explore these impacts across a range of NO_x_ and VOC regimes.

## Methods

In order to explore the nature of the primary radical on ozone production photochemistry the Dynamically Simple Model of Atmospheric Chemical Complexity (DSMACC) zero-dimensional “box” model has been used.^18^ This approach has the advantage of allowing a detailed treatment of the organic oxidation chemistry, at the expense of a comprehensive representation of dynamical processes. A major challenge with the study of tropospheric chlorine oxidation chemistry is the lack of available kinetic and mechanistic data on the relevant reactions. In order to ensure that the modelled differences between Cl and OH oxidation are due to real differences in the chemistry of the two radical species, and not due to missing reactions in the chlorine mechanism, the initial simulations were all performed using methane as the only organic emitted. Methane was selected as the relatively simple oxidation chemistry enables a near explicit mechanism to be used for both the Cl and OH oxidation pathways. Model methane concentrations were chosen to provide organic OH reactivities comparable to those in the troposphere (0.1–20 s^–1^) in order to ensure the balance of organic vs inorganic radical reactions was representative. This is described in more detail in the model validation section in the supplement. The model chemistry scheme is based on the Master Chemical Mechanism (MCM) v3.3.1, with the additional reactions shown in Table S1 to represent Cl oxidation of methane and its oxidation products.^19−22^ Following the methane simulations, the impact of hydrocarbons with different relative OH and Cl reactivities was investigated using simulations of propane and propene. As with the methane simulations, the MCM was used as the base mechanism, with the reactions shown in Table S2 used in addition to those in the methane simulations to represent the chlorine chemistry. While the most relevant reactions of Cl have been included for propane and propene, there are some reactions that could not be included due to a lack of kinetic observational data (e.g., the fate of the chlorinated organics produced through Cl addition products of reactions with propene), so these simulations should not be considered as reliable as those with methane.

As oxidation chemistry controls the losses for both organics and NO_x_ within the model, both primary hydrocarbons (methane, propane, propene) and NO_x_ (as NO) are constrained via a fixed emission, rather than a fixed concentration. This more “realistic” representation of the production of atmospheric NO_x_ and hydrocarbons enables the impact of the radical chemistry on their concentrations to be evaluated. Physical losses of compounds (e.g., mixing or deposition) are represented through a first-order physical loss term, equivalent to a lifetime with respect to physical loss of 24 h. The conclusions of the model simulations are not sensitive to this parameter (see Figure S3), but its use prevents an unrealistic accumulation of oxidation products within the model. Clear sky photolysis rates are calculated using the Tropospheric Ultraviolet and Visible Model (TUV) for July 1^st^ in Los Angeles,^23^ with a surface albedo of 0.1. The model temperature was fixed at 298 K, with a pressure of 1013 hPa and a water vapor concentration of 1%. In all simulations, the model was initialized at local midnight and run for 48 h, with the first day used as a model spin up, and the 12 h centred around solar noon on the second day used for analysis.

Due to the nature of known tropospheric Cl atom sources, the impact of Cl oxidation is likely to be most significant in the morning, when Cl source compounds such as nitryl chloride (ClNO_2_) which have built up in concentration overnight are photolysed the following day. In order to simulate this temporal dependence, primary radicals (both Cl and OH) are produced within the model through a source of ClNO_2_. To isolate the impact of the primary radical (OH vs Cl) on ozone production photochemistry from the impact of NO_x_ on ClNO_2_ production, a fixed ClNO_2_ profile (Figure S2) has been used. This profile represents the higher end of ClNO_2_ concentration profiles observed during the 2010 CalNex campaign,^24^ thus providing a realistic Cl source into the model. In order to assess the difference between OH and Cl oxidation, in the Cl simulations the ClNO_2_ photolysis products are Cl + NO_2_, and in the OH simulations the products are OH + NO_2_. As ClNO_2_ can also react with OH to produce HOCl, in the OH simulations the photolysis and reaction products of HOCl are changed to produce OH instead of Cl and HO_2_ instead of ClO where applicable (see Updates to the gas phase chemistry scheme in the SI).

## Results and Discussion

**Figure 2:**
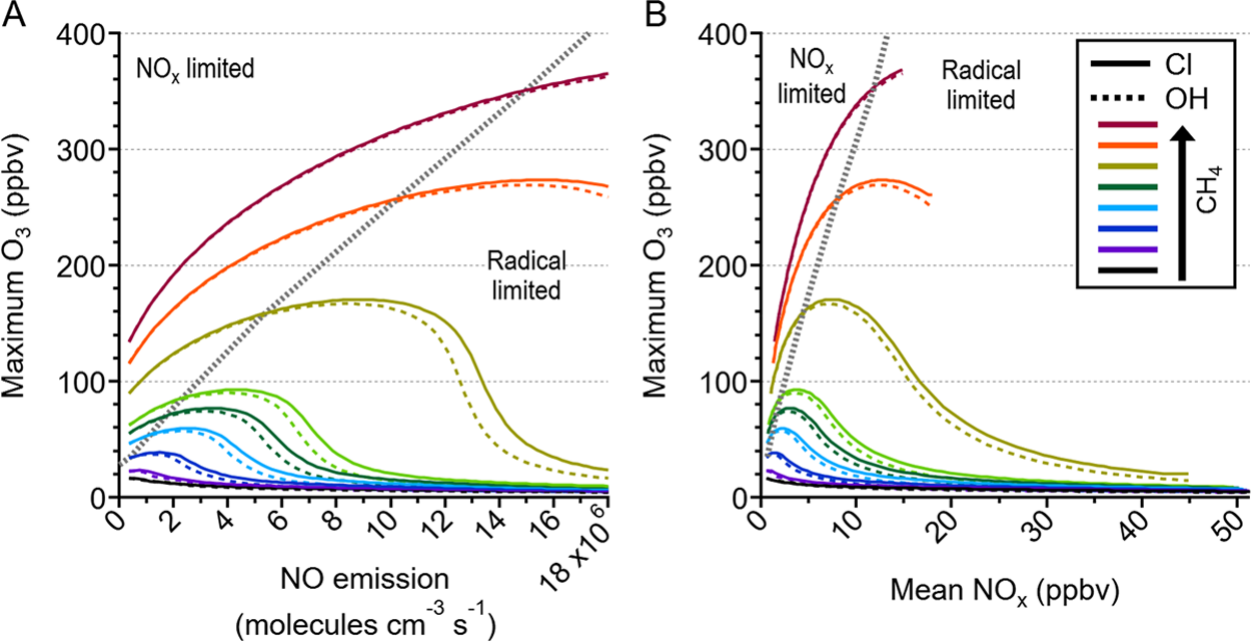
Daily maximum ozone (O_3_) for reactions initiated with Cl (solid lines) and OH (dashed lines) as a function of (a) NO emission and (b) mean NO_x_ for different levels of methane (CH_4_), indicated by different colors. The dashed gray lines indicate the point at which peroxy radical fate via radical–radical reactions is equal to radical-NOx reactions, and thus represents the transition between NOx and radical limitation. The increased NOx losses as model hydrocarbon levels increases results in reduced NOx concentrations as hydrocarbon emissions increase for the same NOx emission, resulting in the change in shape and apparent truncation of the traces between (a) and (b).

The fundamental differences in Cl and OH reactivities may impact atmospheric organic oxidation in multiple ways. In order to investigate these differences, we used model simulations of the simplest hydrocarbon, methane (CH_4_). As ozone is a product of tropospheric organic oxidation, Figure 2a shows simulated daily peak ozone mixing ratios as a function of model NO emission for a range of methane emissions for both the OH (dashed) and Cl (solid) cases. As expected, increasing methane emission leads to increased peak ozone for both OH and Cl. At a given methane emission, peak ozone also increases with increasing NO emission up to the point where the system transitions from NOx-limited to radical-limited ozone production. This transition occurs at the point where the dominant radical fate changes from radical–radical reactions (in NO_x_-limited regime) to reaction with NO_x_ (radical-limited regime). Beyond this point, the rate of increasing ozone production rate with NO emissions reduces (Figure S7) due to greater radical termination, and thus reduced radical propagation efficiency, and the rate of ozone loss to reaction with NO continues to increase, resulting in a maximum in calculated peak ozone mixing ratio followed by a decrease. Under NO_x_-limited conditions, we observe that at a given methane and NO emission the modelled peak ozone is slightly lower (<1%) for the Cl case compared with the OH under NO_x_-limited conditions (Figure S8 shows a zoomed in version of this region of Figure 2a). As the system moves into radical-limited conditions, the Cl case results in increasingly higher peak ozone compared to the OH case (Figure 2a), reaching up to approximately 40% for the high organic and NOx simulations.

Although the absolute magnitudes of the changes in simulated peak ozone are difficult to compare, due to changing non-ClNO_2_ radical sources across the modelled variable space, the relative changes between similar OH and Cl simulations indicates changes in the oxidation of organics within the model driven by the different chemistries of the two radicals. The observed difference in impact on organic oxidation, and thus ozone production, between OH and Cl at any given methane/NO emission is primarily driven by three mechanisms: (i) the relative fraction of radical production that comes from the ClNO_2_ source within the model; (ii) impact of NO_x_ reservoirs; and (iii) relative radical reactivity to organics versus inorganics, which roughly parallels radical propagation versus loss. The fraction of total primary radical production that comes from the ClNO_2_ source within the model changes significantly across the NOx/VOC space explored in these simulations. This is an inevitable consequence of the fact that the magnitude of several primary sources of OH change with the amount of oxidation within the model (e.g., ozone and formaldehyde photolysis, Figure S5). As such, when primary OH production is low the fraction of total radicals coming from the photolysis of ClNO_2_ is large and any difference between the OH and Cl chemistries impacting ozone production will be proportionally larger than if the ClNO_2_ radical source is small compared with other primary OH sources. This effect makes the apparent effect of Cl compared with OH look comparatively lower at high VOC and NOx, meaning that although the relative impact of OH or Cl can be clearly seen in Figure 2, the absolute difference in peak ozone shown is not comparable across the simulated organic/NOx space. As the focus of this work is the different responses of the chemistries of Cl and OH across a representative organic/NOx range, the absolute ozone differences shown in Figure 2 are not important for our conclusions only the direction of change, however, more detail on the changing radical sources across the chemical space explored is provided in the SI.

The impact of NO_x_ reservoirs is driven by the presence of additional NO_x_ reservoir species in the Cl simulations (primarily ClONO_2_ and ClONO), resulting in a reduction in the NO_x_ mixing ratio in the Cl case compared to the OH for a given NO emission. Figure 2b shows modelled peak ozone as a function of mean NO_x_ mixing ratio instead of NO emission, thus removing the effect of the changing NO_x_ reservoir on NO_x_ mixing ratio within the model. The increased NOx losses at higher organic loadings results in the apparent truncation of the simulated ozone at high NOx in the higher methane emission simulations. The remaining difference between the OH and Cl simulations in Figure 2b is due to the relative organic vs inorganic reactivities of the two primary radicals, which becomes the dominant effect in radical limited systems.

For both OH and Cl, virtually all radical reactions with organics lead to radical propagation via the formation of organic peroxy radicals following either hydrogen abstraction or addition to a double bond. However, the inorganic reactions for the two primary radicals in this study differ significantly. The dominant inorganic reaction for OH is reaction with NO_2_ to form the stable species HNO_3_, thus representing a radical sink (R1). In contrast, the dominant inorganic reaction for Cl is with ozone to form the radical species ClO (R2), representing a radical propagation reaction that can result in the recycling of Cl following the reaction of ClO with NO. The reaction of Cl with NO_2_ can also be significant, yielding ClONO (R3a) or ClNO_2_ (R3b), but as both products undergo rapid photolysis to regenerate the Cl and NO_2_ reactants this is largely a null cycle, in contrast to the OH case. The dominant radical termination reaction in the chlorine case is the subsequent reaction of ClO with NO_2_ to produce chlorine nitrate (ClONO_2_, R4).
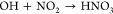
R1
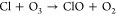
R2
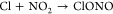
R3a
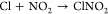
R3b
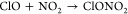
R4

**Figure 3:**
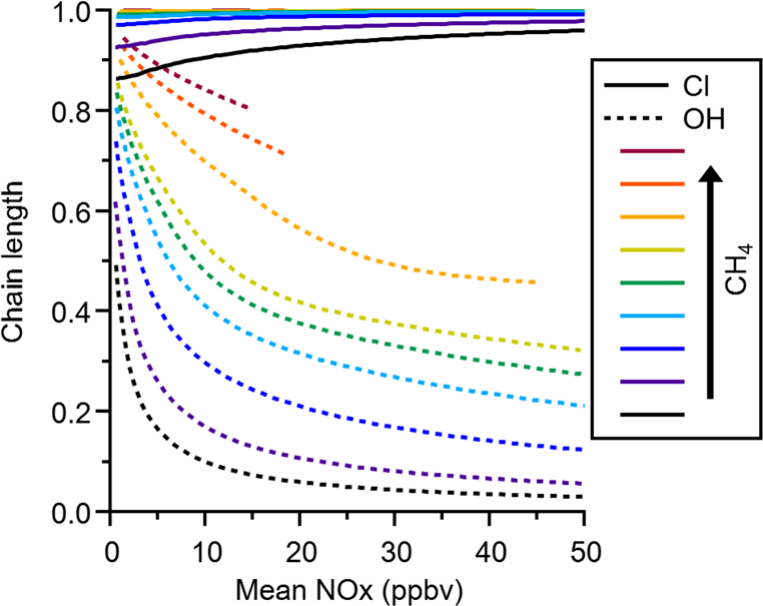
Organic reaction fractions for Cl (solid lines) and OH (dashed lines) in simulations with varying concentrations of methane (CH_4_), indicated by different colors.

Figure 3 shows the organic reaction fractions for OH and Cl for the range of methane and NO emissions modelled, and shows the Cl organic reaction fraction approaching unity at the higher methane concentrations, and tending to unity at higher NO_x_ at the lower methane levels, due to reductions in the ozone concentrations. The OH organic reaction fraction in contrast tends to a value of zero as NO_x_ increases, especially at the lowest methane levels.

**Figure 4:**
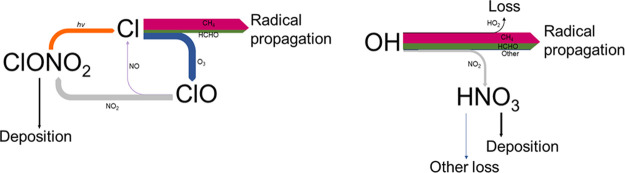
Dominant reaction pathways predicted by the model for Cl (left) and OH (right) under NO_x_-limited conditions. The size of the arrows indicates the probability of the pathway.

Figure 4 shows the dominant reaction pathways for Cl and OH within the model in a NO_x_-limited ozone production regime (NO_x_ mixing ratio = 2 ppbv). In these conditions, the dominant fate for both OH and Cl is reaction with organics, resulting in radical propagation and ozone production. Radical losses via reaction with NO_x_ are small, and the dominant action of NO_x_ is to catalyse radical propagation through the reaction of NO with peroxy radicals. Thus, the reduction in NO_x_ concentration through the production of an additional NO_x_ reservoir (i.e., ClONO_2_), following Cl reaction with ozone to produce ClO, results in a slightly higher ozone production efficiency for OH compared to Cl for equivalent NO emissions (Figure 2a, Figure S8). This impact is likely larger in the real atmosphere, as the simplistic physical loss used in the model may underestimate ClONO_2_ losses to deposition and heterogeneous uptake and thus overestimates the Cl recycled through ClONO_2_ photolysis. A sensitivity simulation was performed where ClONO_2_ physical loss was increased by a factor of 10, resulting in up to a 13 % reduction in peak ozone in the most NO_x_-limited Cl simulations compared to the OH simulation with a comparable NO emission. These differences between OH and Cl in the NO_x_-limited regime are predominantly due to the impact on NO_x_, and are negligible if viewed as a function of NO_*x*_ mixing ratio (Figure 2b) instead of NO emission (Figure 2a).

**Figure 5:**
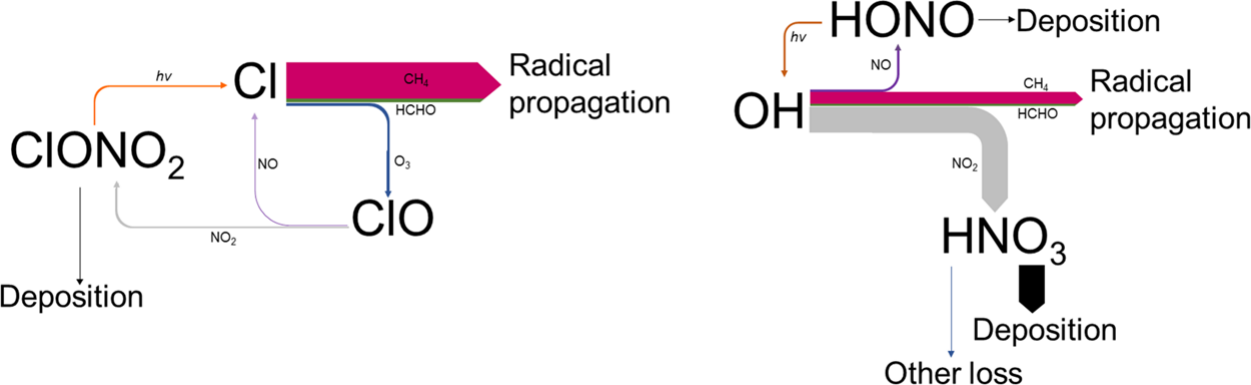
Dominant reaction pathways predicted by the model for Cl (left) and OH (right) under radical-limited conditions. The size of the arrows indicates the probability of the pathway. Despite the higher NO_x_ conditions in this simulation the rate of Cl + NO_2_ is still approximately 0.6 that of the Cl + O_3_ reaction due to the high O_3_ mixing ratio copared with NO_2_ and is thus not included in this figure.

If the model NO_x_ increases sufficiently that the system transitions into a radical-limited regime, the difference between the model peak ozone in the two simulations begins to diverge further, with Cl becoming the more efficient organic oxidizing primary radical. Figure 5 shows the dominant reaction pathways for Cl and OH within the model in a radical-limited ozone production regime (NO_x_ mixing ratio = 19 ppbv). In radical-limited systems NO_x_ reaction is the dominant peroxy radical reaction pathway. The reduction in NO_x_ concentration brought about by additional NO_x_ reservoirs in the Cl case now acts to increase the peak ozone concentration relative to the OH case for the same NO emission. This effect is enhanced if the physical removal of ClONO_2_ within the model is increased.

The ability of a primary radical to initiate oxidative cycles depends on the number of radical reactions that lead to radical propagation versus radical loss. In contrast to the NO_x_-limited regime, where the majority of radical reactions are with organics, the organic oxidation is more sensitive to the relative organic versus inorganic reactivities of the primary radicals in radical-limited systems. Although the rate of inorganic reaction for Cl (R2) is ∼1.2 times faster than that of OH (R1), Cl also reacts ∼16 times faster with methane than does OH (Table S3). Thus, the ratio of radical propagation to loss remains higher for Cl. In relation to ozone production, this is further compounded by the fact that, as NO_x_ increases, the loss of ozone to reaction with NO reduces the ozone concentration and increases the NO_2_ concentration, and thus reduces the Cl inorganic reaction rate (R2) while increasing the OH inorganic reaction rate (R1). Although this does increase the rate of Cl loss to NO2 (R3), the rapid photolysis of the products to reform Cl mean this reaction is of minor importance in the simulations shown here aside from the highest NOx and lowest organic simulations where ozone is reduced significantly. Also, the fates of the inorganic reactions for OH and Cl differ. Unlike HNO_3_ (product of R1) which is relatively unreactive and primarily lost to deposition, the radical species ClO (product of R2) reacts rapidly with NO and HO_2_ to regenerate Cl, or produces ClONO_2_ which can then photolyse to regenerate a fraction of the Cl. Thus, as the system transitions into a radical-limited regime the inorganic loss for OH increases, reducing the efficacy of OH to initiate oxidative cycles. In contrast to Cl, where the reactions remain dominated by organics.

**Figure 6:**
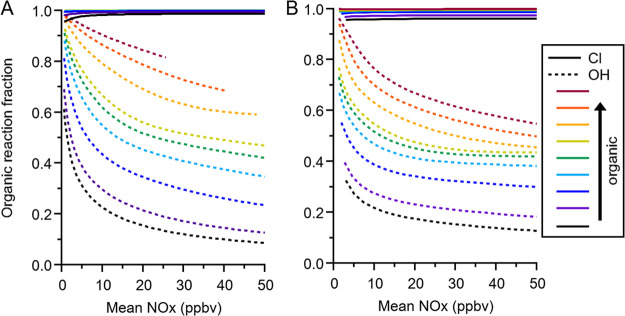
Organic reaction fractions for Cl (solid lines) and OH (dashed lines) in simulations with varying concentrations of (a) propane and (b) propene. The relative reaction rates for the two primary radicals in these simulations are shown in Table S3.

The nature of the organics present also impacts the primary radical effectiveness for hydrocarbon oxidation, as it determines the relative reactivities of the radical plus organic reactions for the different primary radicals (OH and Cl). Chlorine reacts faster with aliphatic carbons than OH, though the extent to which this is the case varies. While biogenics and carbonyls typically react several times faster with Cl than OH, reactions with Cl are usually more than an order of magnitude faster than OH for alkanes, alcohols, alkenes, and substituted aromatics. In the case of methane, explored above, its rate coefficient with Cl is approximately 16 times higher than its rate coefficient with OH (see Table S3). Exploring other organics that have different relative Cl and OH reactivities could be useful, though we are limited in those that can be near-explicitly modelled for Cl chemistry. We chose to additionally examine propane (k_Cl_/k_OH_ = 127) and propene (k_Cl_/k_OH_ = 8.9). For propane, the high organic reactivity for Cl compared with OH further enhances the radical propagation reactions relative to termination for Cl, and thus results in more efficient organic oxidation and thus ozone production in the Cl case under radical-limited regimes (Figures S9, S10). Figure 6 shows the calculated Cl and OH organic reaction fractions for simulations similar to those shown in Figure 3, but for propane (A) and propene (B). In both of these sets of simulations, the simulated OH reactivities of the organics in the model is in a similar range to those in the methane simulations. As in the methane simulations, the propane simulations show an increased efficiency for organic oxidation under radical limited conditions in the Cl case compared to the OH case. However, the effect is enhanced for propane due to the larger difference in the radical + propane rate coefficients meaning that for Cl the organic reaction fractions rapidly approach unity for all propane emission levels. This is similar in the propene simulations, although the OH organic reaction fractions do not tend to zero as quickly as for methane or propane, reflecting the significantly faster rate of OH reaction via addition to the propene carbon–carbon double bond, compared to a Cl reaction rate that is comparable to that with propane (Table S3). This faster OH reaction rate results in a larger fraction of OH reacting with organics for propene compared to propane, and a corresponding decrease in the fraction of OH reacting with NO_2_. The reason that the Cl organic reaction fractions for the lower propene emission scenarios do not reach unity is due to the fact that the propene emissions were scaled to achieve OH reactivities in a similar range to those in the methane simulations. The faster OH reaction rate thus results in a significant decrease in the concentration of organics in the propene case compared with the propane, and hence a reduction in the organic Cl reactivity fraction. As the increased organic oxidation from OH in the propene case ensures ozone production remains high, there is still a significant inorganic sink for Cl thus reducing the Cl organic reaction fraction compared with propane. Another difference between Cl and OH is the regioselectivity of their reactions with organics. For example, in the propane simulations, reactions at the secondary carbon make up 73.6 % of OH reactions and 59 % of Cl reactions. The different regioselectivity could lead to different carbonyl products formed from reactions initiated with Cl and OH, which, through their photolysis and subsequent radical formation, could lead to different in radical abundances. We observed that there were some differences between the radical contributions of carbonyl oxidation product photolysis between Cl and OH (Figure S6). However, the overall contributions of carbonyl photolysis to the radical budget are small, and due to both the regioselectivity of the radicals but also the overall level of organic oxidation. Although these differences were minor relative to other differences between Cl and OH in the simulations discussed above, the effect of this regioselectivity on carbonyl production and subsequent photolysis could be more significant for other organics but the kinetic and mechanistic data to accurately evaluate this in models is currently not available.

## Conclusions and Atmospheric Implications

The model experiments presented in this work show how fundamental chemical differences between OH and Cl can impact tropospheric chemistry. The nature of the primary radical in tropospheric oxidation can impact the efficiency with which organics are oxidized and secondary pollutants are formed. This is determined by the relative fates of the primary radicals, either via radical termination reactions, typically with inorganic species, or radical propagation, typically via reaction with an organic. For the dominant tropospheric primary radical OH, this balance is between its reaction with organics and its major inorganic sink NO_2_. Under NO_x_-limited conditions, OH predominantly reacts with organics, giving a high primary radical effectiveness. As NO_x_ increases this effectiveness reduces as more OH is lost to NO_2_. For Cl primary radicals, the high Cl organic reactivity and fact that much of the Cl that reacts with its inorganic sink is rapidly regenerated, means that organic reactions generally dominate Cl loss across the range of NO_x_ concentrations providing sufficient organics are present. This means that oxidation efficiencies are greater for Cl under radical limited conditions, but similar to OH for NO_x_-limited regimes. This difference can be explained by considering the radical organic reaction fractions of OH and Cl, which tends to 0 for OH with increasing NO_x_ and to 1 for Cl. The rate at which the radicals approach their high NO_x_ organic reaction fraction limit is to a large extent determined by the organic reactivity, and thus the difference in organic oxidation efficiency between the two radicals scales with the difference between their organic reactivities.

Here we considered simple systems with a single initial hydrocarbon, while in the real atmosphere organic mixtures are highly complex. In these systems, the relative efficiency of organic oxidation by Cl compared with OH is dependent on both the relative organic reactivities for each radical, and the relative inorganic radical termination reactions. For example, diurnally-averaged 30 min Cl and OH reactivities were calculated for the 2010 CalNex study in Los Angeles,^9^ where Cl reactivity ranged from 94.9 to 151.7 s^–1^, with radical propagation accounting for 83-91 % of that reactivity (Figure S11). In contrast, OH reactivity was 9.9 to 15.3 s^–1^, with radical propagation accounting for 51-72 % of the reactivity. As the majority of the Cl that reacts with inorganics during the day is recycled, the Cl organic reaction fraction during this study was likely close to 1, compared with approximately 0.67 for OH. This is consistent with Los Angeles being in the radical-limited ozone production regime during the CalNex study,^25,26^ where we expect Cl to be more effective at oxidizing hydrocarbons, and thus producing secondary pollutants, than OH. Most urban environments are radical limited the majority of the time,^27,28^ meaning Cl is currently more effective at producing secondary pollutants in those locations than OH. This impact could be further enhanced by the fact that formation of the primary Cl source in urban environments, ClNO_2_, is dependent on N_2_O_5_ uptake to chloride containing particles, and so generally scales with NO_x_ concentration.^29^ Without detailed chlorine chemistry within atmospheric chemistry models this impact may be underestimated.

In urban areas with high NO_x_ levels that are radical-limited, we expect the relative importance of Cl as a primary radical to be high. This will be most significant in locations with elevated particulate chloride that can be liberated in the form of photolabile Cl atom precursors, such as coastal megacities in areas without strong NO_x_ controls. Mid-continental locations could also see a disproportionately large impact from Cl oxidation if they have a source of chloride that can be liberated from the particle phase. For example, Delhi has high NO_x_ levels and falls in the radical-limited regime, combined with a potentially significant particulate chloride source from waste burning.^30,31^ In many urban areas, emission controls are resulting in declining NO_x_ emissions, leading to a transition from radical-limited to NO_x_-limited regimes.^32,33^ This would likely result in decreasing impact of primary radical oxidation from Cl relative to OH. In areas that are transitioning toward NO_x_-limited regime and have a source of Cl, it is also probable that Cl had higher impacts relative to OH in the past. One example in which this could have been the case is Los Angeles during the mid-to-late 1900s when both NO_x_ and O_3_ levels were very high.^25,34^ These effects would not have been present in the models used to advise policy during that time due to a lack of inclusion of Cl chemistry. Ultimately, for models to accurately predict the response of secondary pollutants to changes in NO_x_ and organic emissions, the nature and detailed chemistry of the primary radicals needs to be considered. Most current models have limited inclusion of Cl-initiated chemistry, particularly reactions of Cl with organics.^8^ More kinetic and mechanistic data is needed for reactions of Cl, as well as Cl-containing products, to allow explicit modelling of even simple organics. For example, the fate of HC(O)Cl, a product of Cl addition to simple alkenes,^13^ is not well understood.^12,14^ The differences between Cl and OH also have implications for any future changes in primary radical sources, such as a decrease in Cl production with decreasing NO_x_ due to reduced ClNO_2_ formation, or the intentional increase in Cl production as a proposed strategy to reduce methane lifetime.^35^

## Supporting Information Available

Additional details on model configuration and validation, including chlorine chemistry updates made to the MCM chemical scheme. Additional model outputs that support the conclusions presented in the paper. (PDF)
