# Supplementary material for: Primary Radical Effectiveness: Do the Different Chemical Reactivities of Hydroxyl and Chlorine Radicals Matter for Tropospheric Oxidation?
Source: ACS EST Air. 2024 May 23;1(8):780–8. doi: 10.1021/acsestair.3c00108 (PMC11320563; doi:10.1021/acsestair.3c00108)
Supplement: Supplementary file 1 — ea3c00108_si_001.pdf [file ea3c00108_si_001.pdf]

Supplemental Information for:

Primary radical effectiveness: Do the different chemical reactivities of hydroxyl and chlorine radicals matter for tropospheric oxidation?

Peter M. Edwards[1,2] and Cora J. Young[3]

[1]Wolfson Atmospheric Chemistry Laboratories, Department of Chemistry, University of York, York, UK
[2]National Centre for Atmospheric Science, University of York, York, UK
[3]Department of Chemistry, York University, Toronto, ON Canada
Correspondence to: pete.edwards@york.ac.uk

Radical reactivity

Radical reactivity for Cl or OH towards any individual chemical is defined as:

$$reactivity_{Cl+X} = k_{Cl+X}[X]$$

$$reactivity_{OH+X} = k_{OH+X}[X]$$

We can also define the total reactivity for a radical or the reactivity of a radical for certain types of reactants. For example, the total reactivity for Cl is defined as the sum of all individual Cl reactivities:

$$reactivity_{Cl,tot} = \sum reactivity_{Cl+X}\,[X]$$

Model set-up

The DSMACC 0-D model was used to investigate the impact of the different chemistries of OH and Cl primary radicals on ozone production as the lack of a detailed representation of physical or heterogeneous processes enables the isolation of the chemical factors. The model uses the Master Chemical Mechanism (MCM v 3.3.1) as its base chemical scheme, but this required significant modification in order to include our best representation of Cl + VOC chemistry. The following sections explain the changes made to the standard DSMACC implementation and mechanism (e.g. Edwards et al. 2011) for this work.

Estimation of J-values

The photolysis rate coefficients (J-values) for $ClNO_2$, Cl_2, ClO and HCl were taken from the TUV model and formatted for incorporation into MCM. Additional photolabile chlorine-containing molecules that are not included in MCM were parameterized as linear combinations of $J(O_3)$ and $J(NO_2)$. Values of $J(O_3)$ and $J(NO_2)$ were calculated using the TUV model at solar zenith angles

of 0, 5, 10, 15, 20, 30, 40, 50, 60, 70, 75, 80, and 85. Using these 12 points, J-values for chlorine-containing molecules were determined by linear combinations of $J(O_3)$ and $J(NO_2)$.

$J(HOCl) = 0.024*J(NO_2) + 1.476*J(O_3)$

$J(ClNO) = 0.220*J(NO_2) + 3.313*J(O_3)$

$J(ClONO) = 0.342*J(NO_2) + 27.89*J(O_3)$

$J(ClONO_2)1 = 0.0034*J(NO_2) + 0.1879*J(O_3)$

$J(ClONO_2)2 = 0.00046*J(NO_2) + 0.1060*J(O_3)$

Updates to the gas phase chemistry scheme

As the MCM only contains a small number of Cl + VOC reaction, primarily Cl + alkane, the chemical mechanism was updated using available literature values in order to achieve as close to an explicit chlorine scheme as possible for the 3 primary hydrocarbons investigated (Methane, propane and propene). Recommended values based on evaluation summaries (e.g. NASA JPL or IUPAC chemical Kinetics databases) were chosen preferentially if available. If evaluation recommendations were not available, then experimental values were used ahead of theoretical calculated values. If no literature values were available, then rates of similar reactions were used. Table S1 summarises the additional chlorine reactions included and the source of the kinetic data.

Table S1. Reactions added to MCM for the simulation of methane chemistry with OH and Cl.

Reactants	Products	Rate coefficient	Reference
CL + O3	CLO	2.8D-11*EXP(-250/TEMP)	[1]
HO2 + CL	HCL	3.5D-11	[2]
HO2 + CL	OH + CLO	7.5D-11*EXP(-620/TEMP)	[1]
CL + H2O2	HCL + HO2	1.1D-11*EXP(-980/TEMP)	[1]
CL + NO3	NO2 + CLO	2.4D-11	[1]
CL + CLONO2	CL2 + NO3	6.2D-12*EXP(145/TEMP)	[1]
OH + HCL	CL	1.7D-12*EXP(-230/TEMP)	[1]
OH + CL2	HOCL + CL	3.6D-12*EXP(-1200/TEMP)	[1]
OH + HOCL	CLO	5.0D-13	[1]
OH + CLO	CL + HO2	1.8D-11	[2]
OH + CLO	HCL	1.2D-12	[2]
CLO + NO2	CLONO2	7.0D-11	[1]
CLO + HO2	HOCL	2.2D-12*EXP(340/TEMP)	[1]
CLO + NO	CL + NO2	6.2D-12*EXP(295/TEMP)	[1]
O + CLO	CL	2.5D-11*EXP(110/TEMP)	[1]
OH + CLNO2	HOCL + NO2	2.4D-12*EXP(-1250/TEMP)	[1]
O + HOCL	CLO + OH	1.7D-13	[1]
CL + H2	HCL + HO2	3.9D-11*EXP(-2310/TEMP)	[1]
OH + CLONO2	HOCL + NO3	1.2D-12*EXP(-330/TEMP)	[1]
O + CLONO2	CLO + NO2	4.5D-12*EXP(-900/TEMP)	[1]
CL + NO2	CLONO	1.3D-30*M*(TEMP/300)**-2	[2]
CL + NO2	CLNO2	1.8D-31*M*(TEMP/300)**-2	[2]
CL + NO	CLNO	7.7D-32*M*(TEMP/300)**-1.8	[2]
HCHO + CL	HCL + HO2 + CO	8.1D-11*EXP(-34/TEMP)	[3]
CH3OH + CL	HO2 + HCHO + HCL	7.1D-11*EXP(-75/TEMP)	[3]
CH3OOH + CL	HCHO + OH + HCL	5.9D-11	[3]
CH3NO3 + CL	HCHO + NO2 + HCL	2.4D-13	[3]

Table S2. Reactions added to MCM (in addition to those listed in Table S1) for the simulation of propene and propene chemistry with OH and Cl.

Reactants	Products	Rate coefficients	Reference
CH3CHO + CL	CH3CO3 + HCL	7.92D-11	[3]
CH3CHO + CL	HCOCH2O2 + HCL	8.0D-13	[3]
C2H5CHO + CL	C2H5CO3 + HCL	1.3D-10	[3]
GLYOX + CL	CO + CO + HO2 + HCL	4.86D-11*EXP(-34/TEMP)	[4]
GLYOX + CL	HCOCO3 + HCL	3.24D-11*EXP(-34/TEMP)	[4]
CH3COCH3 + CL	CH3COCH2O2 + HCL	1.5D-11*EXP(-590/TEMP)	[3]
C2H5OH + CL	CH3CHO + HO2 + HCL	5.5D-11*EXP(155/TEMP)	[3]
C2H5OH + CL	HOCH2CH2O2 + HCL	4.8D-12*EXP(155/TEMP)	[3]
NPROPOL + CL	C2H5CHO + HO2 + HCL	1.6D-11*EXP(525/TEMP)	[3]
NPROPOL + CL	HO1C3O2 + HCL	4.1D-12*EXP(525/TEMP)	[3]
NPROPOL + CL	HYPROPO2 + HCL	6.8D-12*EXP(525/TEMP)	[3]
IPROPOL + CL	CH3COCH3 + HO2 + HCL	7.4D-11	[3]
IRPOPOL + CL	IPROPOLO2 + HCL	1.3D-11	[3]
ETHGLY + CL	HOCH2CHO + HO2 + HCL	2.5D-10	[4]
PROPGLY + CL	ACETOL + HO2 + HCL	1.26D-10	[4]
PROPGLY + CL	CH3CHOHCHO + HO2 + HCL	7.94D-11	[4]
HCOOH + CL	HO2 + HCL	1.9D-13	[3]
CH3CO2H + CL	CH3O2 + HCL	2.65D-14	[3]
PROPACID + CL	C2H5O2 + HCL	3.96D-14	[4]
C2H5NO3 + CL	CH3CHO + NO2 + HCL	4.7D-12	[3]
NC3H7NO3 + CL	C2H5CHO + NO2 + HCL	2.2D-11	[3]
IC3H7NO3	CH3COCH3 + NO2 + HCL	3.8D-12	[3]
IPROCLO2 + HO2	IPROPOLO2H	KRO2HO2*0.520	Formed from the addition of Cl + C_3H_8; analogous to the MCM OH peroxy radical, IPROPOLO2.
IPROCLO2 + NO	IPROPOLO + NO2	KRO2NO*0.991	
IPROCLO2 + NO	PROLNO3	KRO2NO*0.009	
IPROCLO2 + NO3	IPROPOLO + NO2	KRO2NO3	

IPROCLO2	CH3CHOHCHO	2.00D-12*0.2*RO2	Assuming same rate and fractionation as IPROPOLO2.
IPROCLO2	IPROPOLO	2.00D-12*0.6*RO2	
IPROCLO2	PROPGLY	2.00D-12*0.2*RO2	
HYPROCLO2 + HO2	HYPROPO2H	KRO2HO2*0.520	Formed from the addition of Cl + C_3H_8; analogous to the MCM OH peroxy radical, HYPROPO2. Assuming same rate and fractionation as HYPROPO2.
HYPROCLO2 + NO3	HYPROPO + NO2	KRO2NO3	
HYRPOCLO2	ACETOL	8.80D-13*0.2*RO2	
HYPROCLO2	HYPROPO	8.80D-13*0.6*RO2	
HYPROCLO2	PROPGLY	8.80D-13*0.2*RO2	
HYPROCLO2 + NO	HYPROPO + NO2	KRO2NO*0.977	
HYPROCLO2 + NO	PROPOLNO3	KRO2NO*0.023	

Table S3. Rate coefficients for selected molecules with Cl and OH at 298K.

**Molecule**	**kCl (cm^3 $molecule^{-1}$ s^{-1})**	**kOH (cm^3 $molecule^{-1}$ s^{-1})**	**kCl/kOH**
Methane	1.0 x 10-13 [2]	6.3 x 10-15 [2]	15.9
Propane	1.4 x 10-10 [2]	1.1 x 10-12 [2]	127
Propene	1.34 x 10-10 [3]	1.5 x 10-11 [3]	12.2
O_3	1.2e-11 [2]		
NO_2		1.39e-11 [2]	

In the OH simulations, where the $ClNO_2$ photolysis products were changed to OH + NO_2, there was a need to also modify the reactions of HOCl due to its production via OH + $ClNO_2$. This represented a negligible change to the model chemistry, but was required to ensure there was no Cl in the OH simulations. The three reactions that were changed are given below, with the OH products in parenthesis.

HOCl + O --> ClO(HO_2) + OH
HOCl + hv --> Cl(OH) + OH
HOCl + OH --> ClO(HO_2)

Model validation

Although the simplified model simulations used in this study are by design unrealistic representations of the real atmosphere, due to a desire to keep the VOC chemistry as simple as possible to enable diagnosis of the source of any differences between OH and Cl oxidation, it is our intention to keep the simulations as realistic as possible within these constraints. The following sections provide supporting detail on the model simulations and their sensitivities.

Model constraints and spin-up.

Model simulations were initialised at 4am local time on July 1st, using the CALNEX ground site (34.140582 N, 118.122455 W) location for TUV calculations, and a surface albedo of 0.1. Temperature and pressure were fixed at 298 K and 1013 hPa respectively, and water vapour was fixed at 1%. All simulations were initialised with 40 ppb of ozone. In order to represent the impact of Cl vs OH chemistry on NOx within the model a fixed first-order emission of NO was used to obtain the targeted NOx concentrations. This enabled the impact of additional NOx reservoir species in the Cl simulations to be investigated and was considered a more realistic representation than fixing the NO and NO_2 concentrations, which would result in changes in the effective NOx emission as NOx reservoir production varied. In order to reduce the spin-up time required to reach a stable NOx concentration, models were initialised with starting concentrations of NO_2 equivalent to 50 pptv for the lowest NOx simulations through to 14 ppbv for the highest. This resulted in average model NOx mixing ratios ranging from approximately 0.5 to 50 ppbv across the 100 different NOx levels simulated for each VOC level (see Figure 2 in main text). Figure S1 shows example NO and NO_2 profiles from different NOx and CH_4 emission simulations. As described in the main text, simulations were performed using single primary VOCs in each simulation (methane, propane or propene) in order to focus on the specific differences in the chemistries, using near explicit mechanisms for both the OH and Cl oxidation pathways. In order to make this simplistic representation of atmospheric VOCs as representative as possible, 10 target VOC concentrations were chosen to cover a realistic range of OH reactivities, ranging from approximately 0.1 s^{-1} in the lowest VOC simulations (corresponding to minimum mixing ratios of approximately 500 ppbv for CH_4, 3 ppbv for C_3H_8, and 0.07 ppbv for C_3H_6) to approximately 20 s^{-1} in the highest VOC simulations (corresponding to maximum mixing ratios of approximately 80 ppmv for CH_4, 800 ppbv for C_3H_8, and 20 ppbv for C_3H_6). This was deemed a preferential approach to using atmospherically realistic mixing ratios of each of the VOCs as this made it difficult to achieve realistic radical levels and thus ozone production within the simulations. As with NOx, the VOCs were constrained using a starting concentration and a fixed first-order emission, scaled to achieve the targeted OH reactivity equivalent VOC concentrations. Simulations were performed across 10 different VOC levels, for each of the 100 NOx levels.

Simulations were run for 48 hours, with the first day used to generate realistic levels of secondary oxidation products, and the 12-hours centred around local solar noon on the second day used for the analysis presented. Figure S1 shows some example model mixing ratio profiles for O_3, NO, NO_2, OH, HO_2 and formaldehyde, showing that atmospherically relevant values for all these species are achieved within the range of variable space covered by the simulations, despite the simplistic single primary VOC approach.

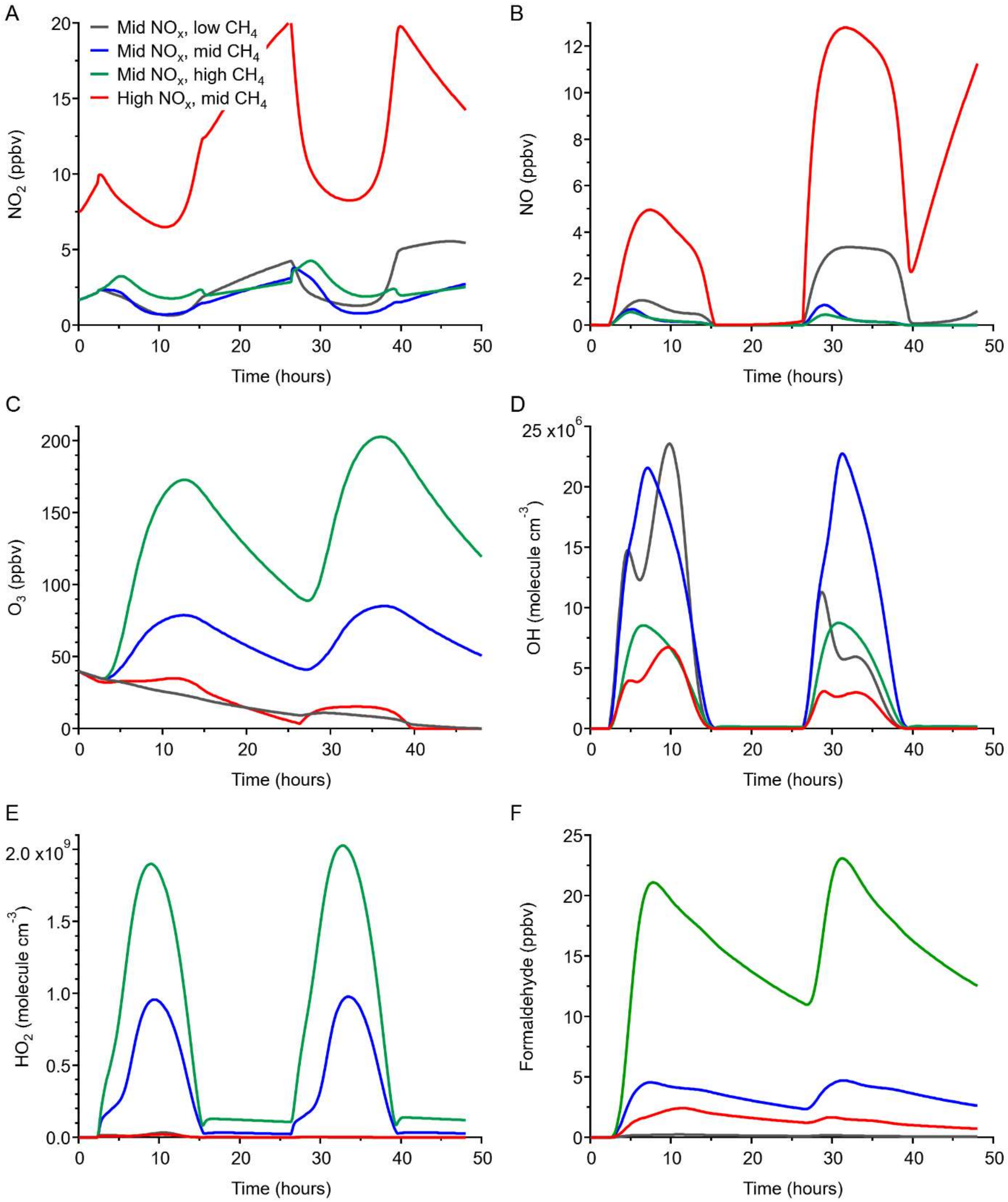

Figure S1: Example methane model simulation outputs for (a) NO_2, (b) NO, (c) O_3, (d) OH, (e) HO_2, and (f) formaldehyde across a range of NOx and CH_4 levels covered by the simulations.

As described in the main text, the only species within the model that was constrained via a fixed diurnal profile was $ClNO_2$. This was done in order to isolate the impact of the different reactions of the OH and Cl chemistries under different NOx and VOC conditions, without the much larger

effect of NOx on $ClNO_2$ production, via N_2O_5 production and uptake. Increasing levels of NOx and O_3 will inevitably result in increased N_2O_5 production and uptake to aerosol, and providing sufficient available particulate chloride this will result in the production of $ClNO_2$. This has been the focus of multiple previous studies (e.g., [5–7]) and is thought to be the primary driver of chlorine chemistry in many coastal urban environments. In this study, however, we are wanting to investigate the impacts of the different reactivity profiles of OH and Cl, not the production mechanisms. For this reason it was decided to use a fixed diurnal profile for $ClNO_2$ (Figure S2), in order to provide direct comparisons between the OH and Cl simulation cases. The diurnal profile chosen was generated by a free running model with parameterised $ClNO_2$ production and tuned to achieve a maximum within the range of the reported $ClNO_2$ concentrations observed during the CALNEX campaign.[8] Simulations where the magnitude of this $ClNO_2$ profile were scaled with model NOx emission were explored, however, the simulated Cl chemistry was highly sensitive to this scaling, as would be expected, and normalising for this effect in order to isolate the impact of the chemistry of interest was deemed to add unnecessary complexity compared with the fixed $ClNO_2$ profile across all simulations.

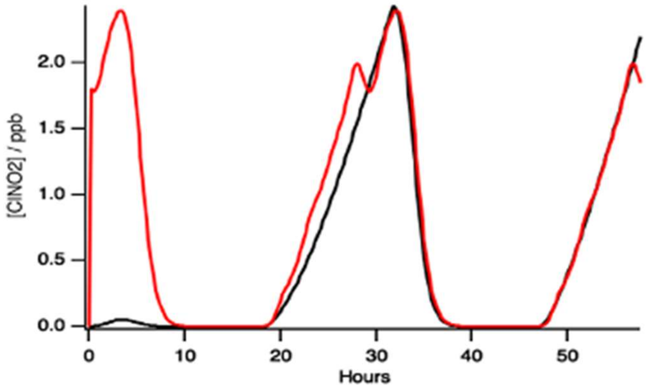

Figure S2: Constrained $ClNO_2$ profile used in all simulations.

In order to represent the physical removal of species from the model (e.g. via deposition or mixing), and prevent the unrealistic build up of oxidation products, a first order loss process was used for all species, with a lifetime of 24 hrs with respect to this loss. As stated in the main text, sensitivity studies were carried out and confirm that our conclusions are not sensitive to this model parameter. Figure S3 shows the simulated ozone for three different methane levels across the full NOx range investigated with the 24 hr lifetime for species to physical removal (solid) and a 12 hr lifetime to physical loss (dashed line).

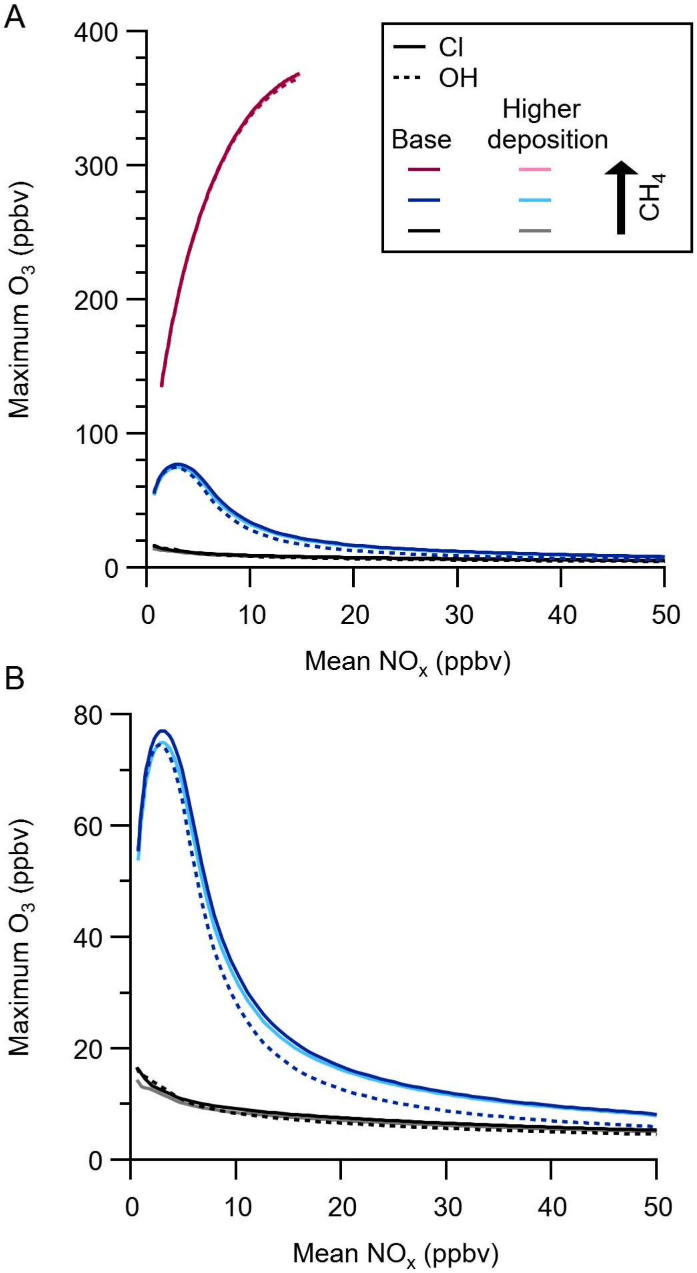

Figure S3. Comparison of simulations using base conditions and increased deposition showing daily maximum ozone for reactions initiated with Cl (solid lines) and OH (dashed lines) as a function of mean NO_x for different levels of CH_4 for (a) full range and (b) lower CH_4 simulations.

Atomic chlorine as a fraction of the total radical budget

Although the $ClNO_2$ source of Cl or OH is constant throughout all the model simulations, the magnitude of other radical sources changes significantly as the VOC and NOx concentrations change. This means that throughout the model variable space explored in this work the fraction

of the total radicals coming from $ClNO_2$ changes. Figure S4 shows the fraction of the total daytime average OH + Cl production that is Cl in the $ClNO_2$ --> Cl + NO_2 simulations, showing a range from 0.1% of the total OH + Cl production in the highest methane and highest NOx simulations to as much as 46% in the lowest methane and highest NOx simulations. This change in the fractional contribution of $ClNO_2$ as a radical source in these simulations makes it difficult to compare the absolute magnitude of the impact of Cl vs OH on ozone production across multiple simulations, such as Figure 2 in the main text, and for this reason our analysis and interpretation focussed on the radical reactivities and in particular the organic reaction fraction. It is worth noting, however, that the largest observed differences in peak ozone between OH and Cl simulations shown in Figure 2 are for the highest methane level that fully transitions into the radical limited regime (orange), for which the fraction of total radical production that comes from $ClNO_2$ is the lowest compared to other simulations in this chemical regime (Figure S4).

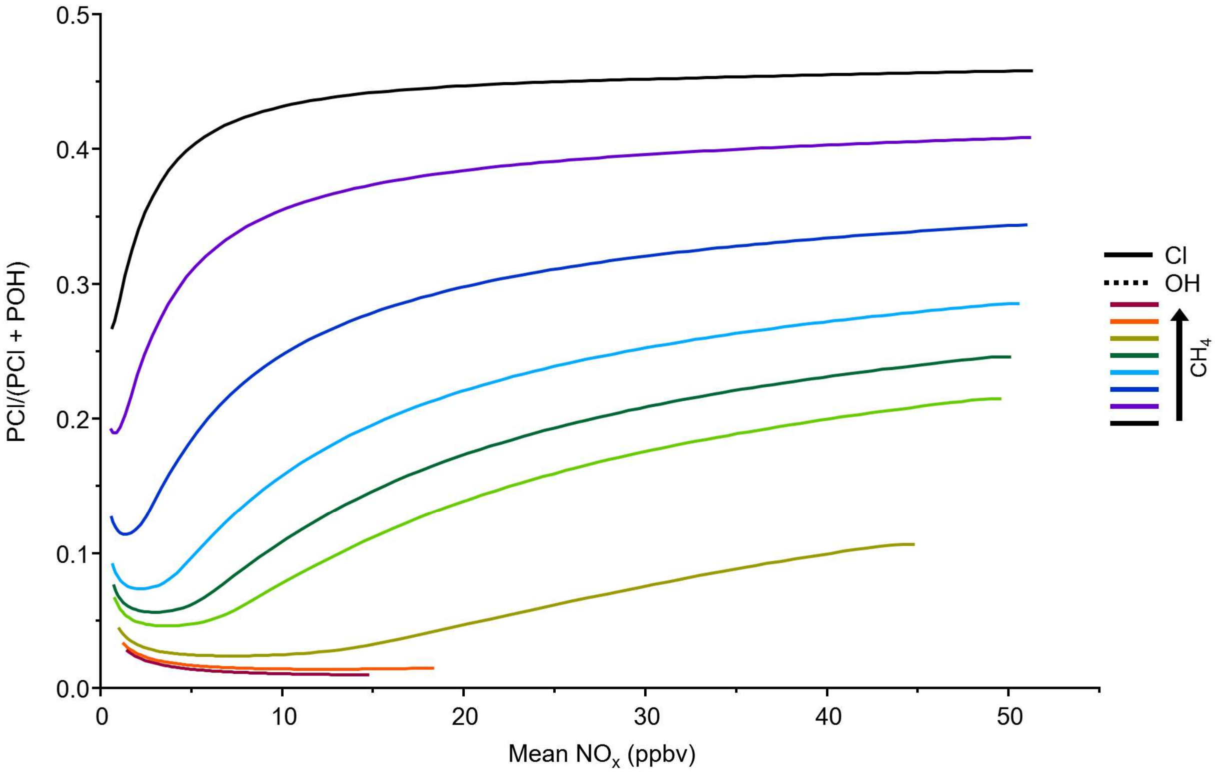

Figure S4: Fraction of daytime average model radical (OH + Cl) production rate that comes from $ClNO_2$ photolysis.

The production of OH within the model comes from both primary and secondary sources, and the relative magnitudes of these change depending on the NOx and VOC concentrations. Figure S5 shows the magnitudes of the significant radical production reactions within the model for 3 simulations with methane in the middle of the range studied and either low, mid or high NOx, in

both the Cl and OH implementations of these simulations. Figure S5A shows that under low-NOx conditions the differences between the OH source strengths are very similar for both the OH and Cl simulations. As NOx increases, and the simulations transition into a more radical limited regime, the OH source from both formaldehyde and ozone photolysis becomes larger in the Cl simulations compared with the analogous OH simulations. This is due to the increased hydrocarbon oxidation from the primary radicals in the Cl simulations, due to the increased radical organic reaction fraction for Cl, resulting in increased production of both formaldehyde and ozone. The secondary OH production from NO + HO_2 is also larger in the mid and high NOx Cl simulations than in the analogous OH simulations, again due to increased hydrocarbon oxidation resulting in more radical propagation in the Cl case and thus greater HO_2 production. Figure S5B shows the same data as in Figure S5A but as a stacked plot, showing the change in total radical source across the NOx range investigated. Figure S6 shows a similar breakdown of model radical sources but for the propane simulations, highlighting the negligible impact of larger oxygenates as radical sources within the simulations.

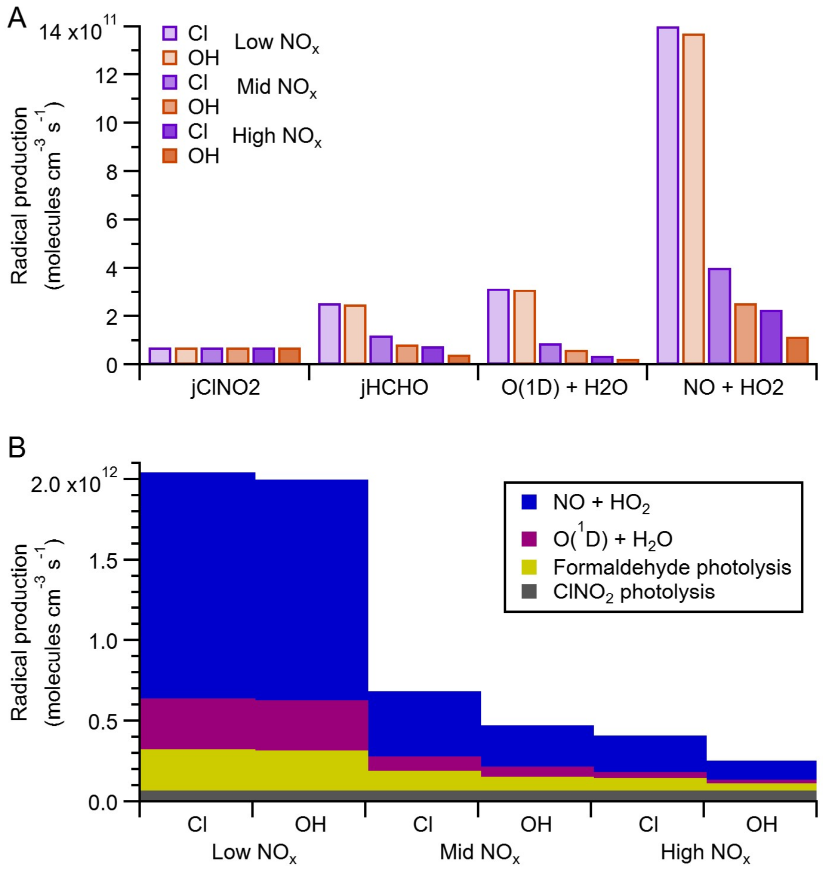

Figure S5: Radical production in methane (CH_4) simulations grouped by (a) radical source; and (b) simulation.

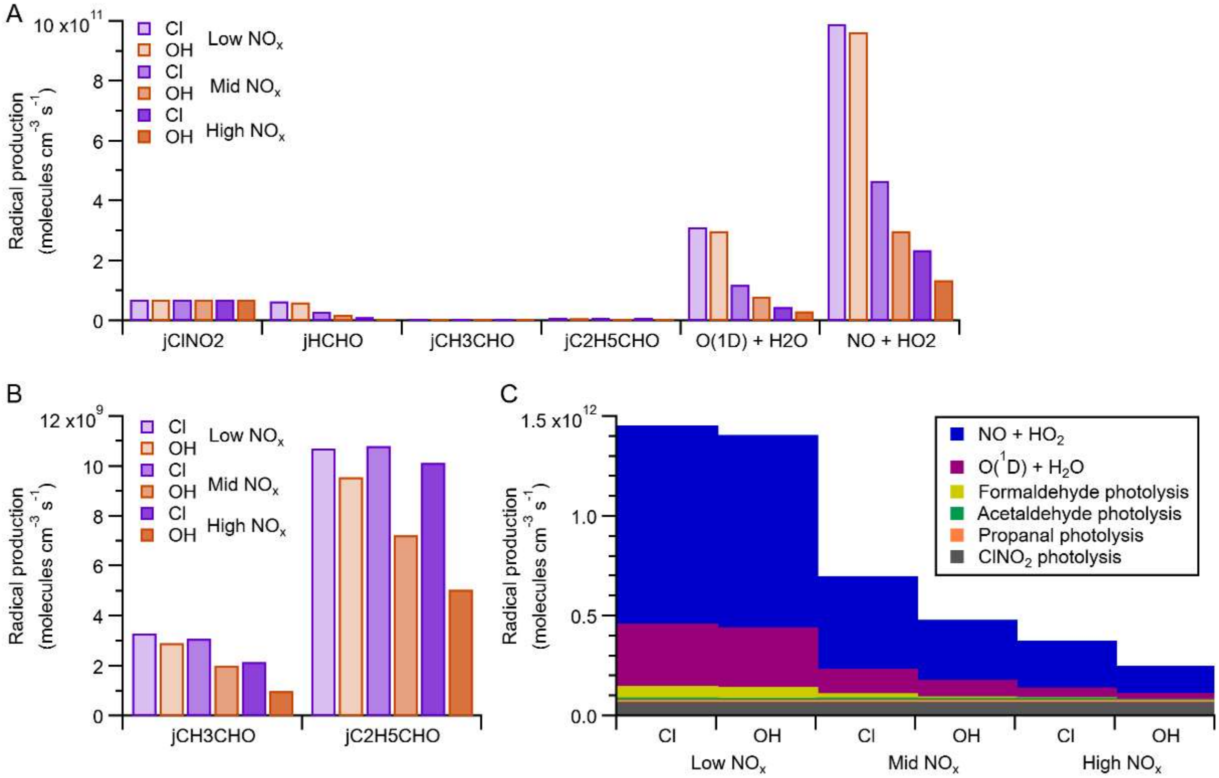

Figure S6: Radical production in propane (C_3H_8) simulations for (a) grouped by radical sources for all major radical sources; (b) acetaldehyde and propanal only; and (c) grouped by simulation.

Additional model outputs

The following plots show additional model diagnostics that, although not central to our conclusions, support the evidence presented in the main text.

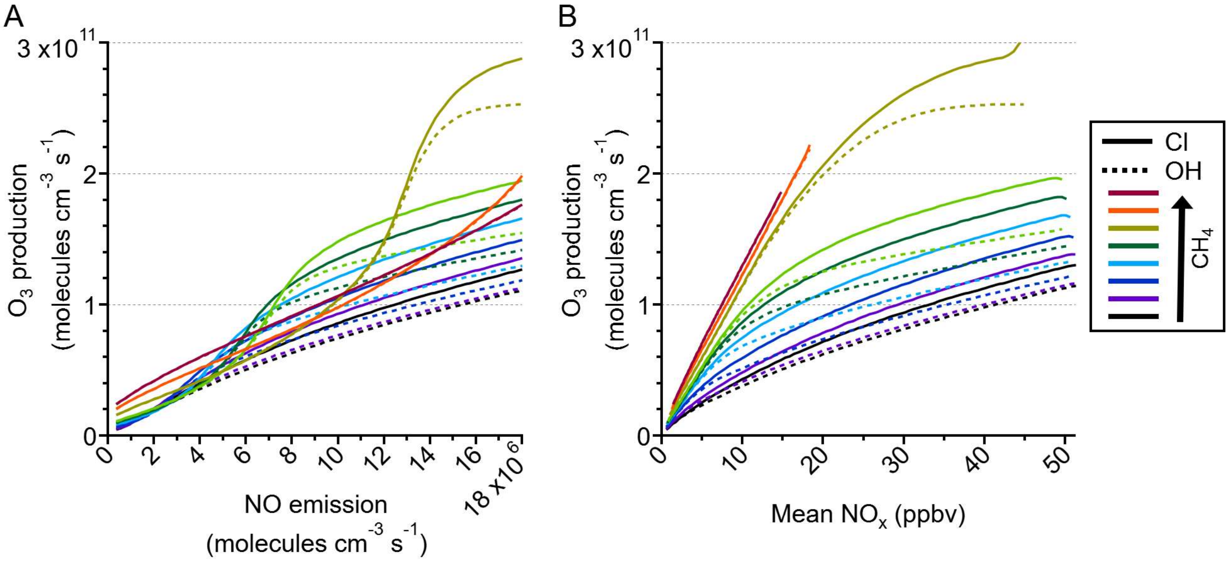

Figure S7. Ozone (O_3) production for reactions initiated with Cl (solid lines) and OH (dashed lines) as a function of (a) NO emission and (b) mean NO_x for different levels of methane (CH_4), indicated by different colours.

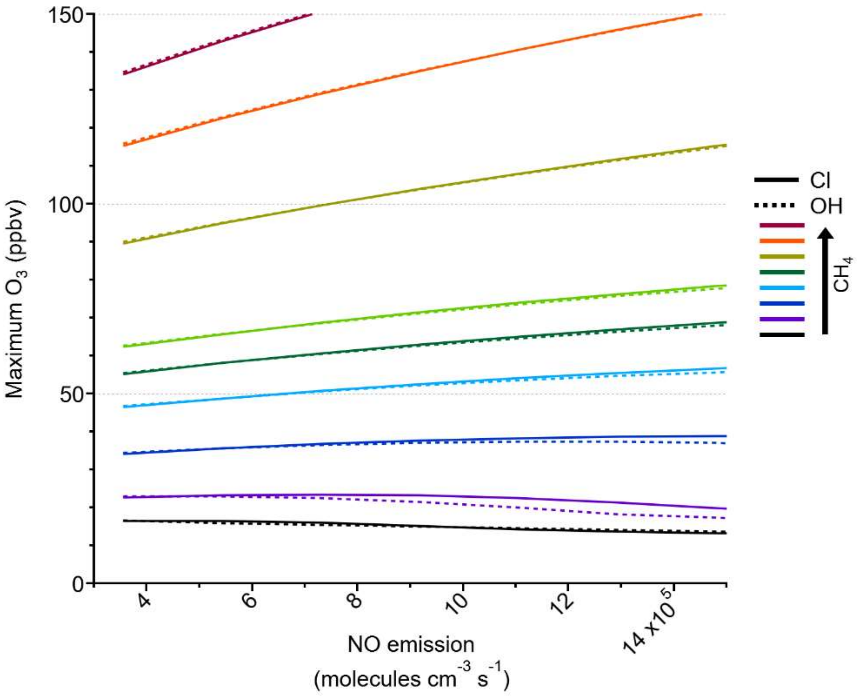

Figure S8. Daily maximum ozone (O_3) for reactions initiated with Cl (solid lines) and OH (dashed lines) as a function of NO emission for different levels of methane (CH_4), indicated by different colours focused on low NO emission regime. Same data as Figure 2a.

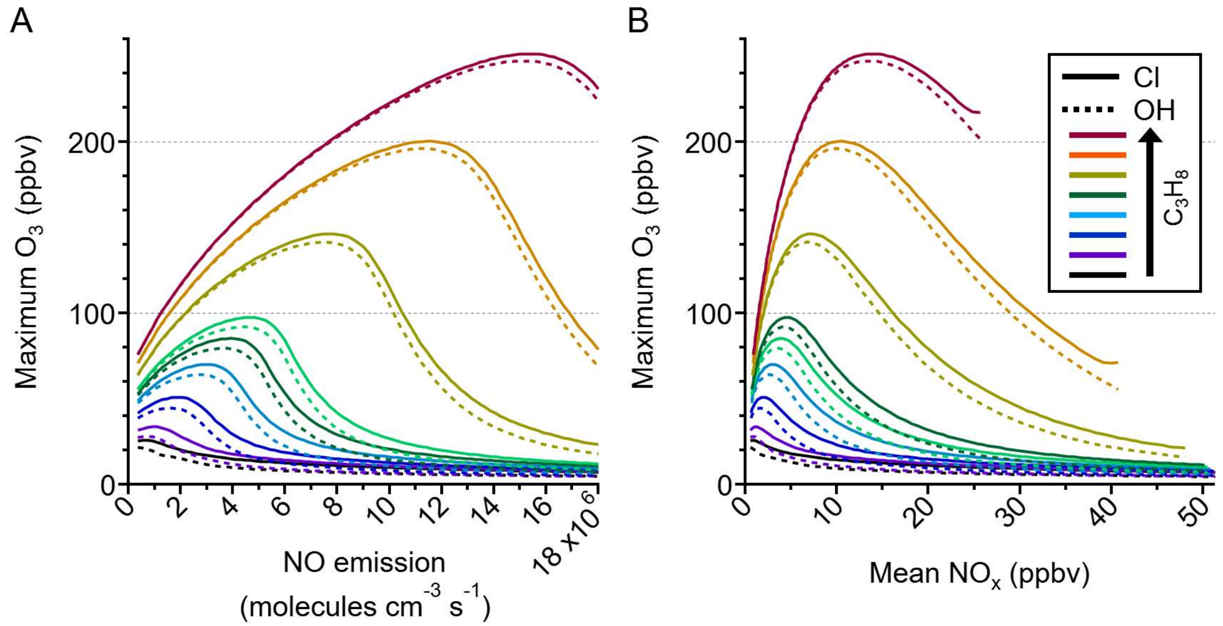

Figure S9. Daily maximum ozone (O_3) for reactions initiated with Cl (solid lines) and OH (dashed lines) as a function of (a) NO emission and (b) mean NO_x for different levels of propane (C_3H_8), indicated by different colours.

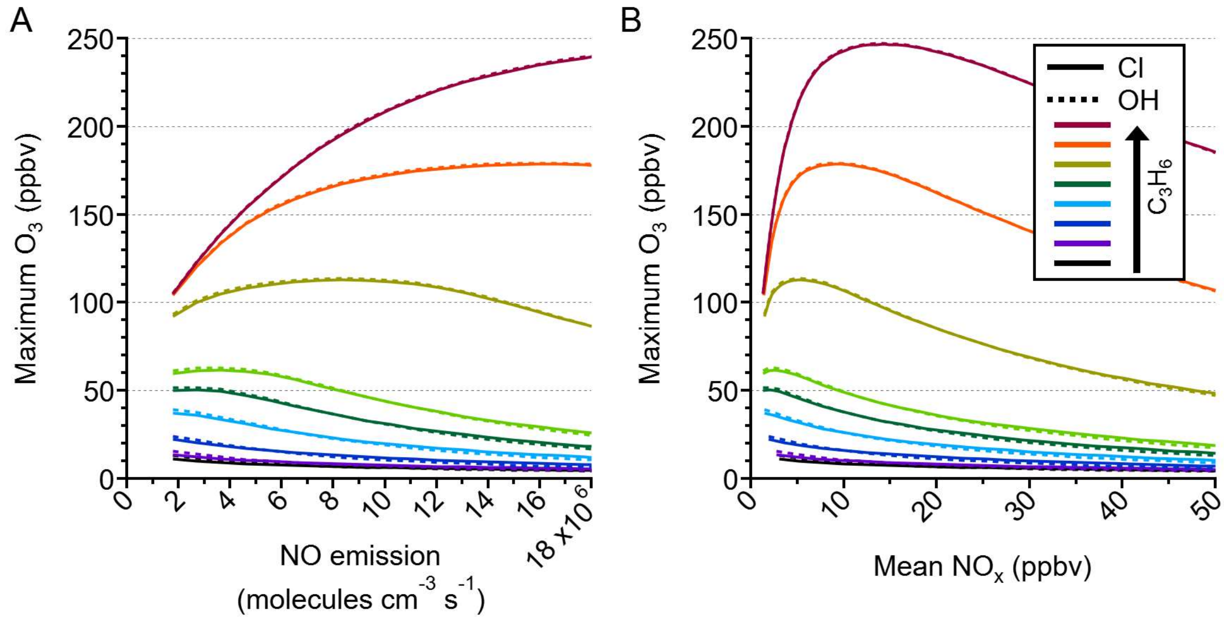

Figure S10 Daily maximum ozone (O_3) for reactions initiated with Cl (solid lines) and OH (dashed lines) as a function of (a) NO emission and (b) mean NO_x for different levels of propene (C_3H_6), indicated by different colours.

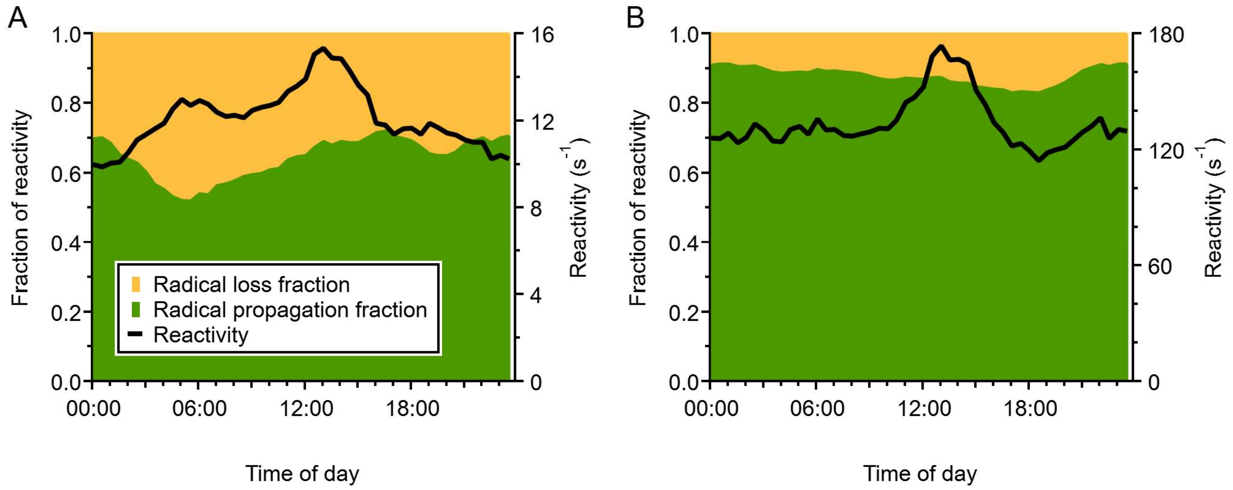

Figure S11. Fraction of radical reactivity attributed to loss versus propagation, shown with total calculated reactivity for (a) OH and (b) Cl during the 2010 CalNex campaign in Los Angeles. Data from Young et al.[9]

References

(1) Atkinson, R.; Baulch, D.; Cox, R.; Crowley, J.; Hampson, R.; Hynes, R.; Jenkin, M.; Rossi, M.; Troe, J. Evaluated Kinetic and Photochemical Data for Atmospheric Chemistry: Volume III - Gas Phase Reactions of Inorganic Halogens. *Atmos Chem Phys* **2007**, *7*, 981–1191.

(2) Burkholder, J. B.; Sander, S. P.; Abbatt, J. P. D.; Barker, J. R.; Cappa, C.; Dibble, T. S.; Hui, R. E.; Kolb, C. E.; Kurylo, M. J.; Orkin, V. L.; Percival, C. J.; Wilmouth, D. M.; Wine, P. H. *Chemical Kinetics and Photochemical Data for Use in Atmospheric Studies Evaluation Number 19*; Jet Propulsion Laboratory: Pasadena, CA, 2019. http://jpldataeval.jpl.nasa.gov.

(3) Atkinson, R.; Baulch, D. L.; Cox, R. A.; Crowley, J. N.; Hampson, R. F.; Hynes, R. G.; Jenkin, M. E.; Rossi, M. J.; Troe, J. Evaluated Kinetic and Photochemical Data for Atmospheric Chemistry: Volume II - Gas Phase Reactions of Organic Species. *Atmos Chem Phys* **2006**, *6*, 3625–4055.

(4) Xue, L. K.; Saunders, S. M.; Wang, T.; Gao, R.; Wang, X. F.; Zhang, Q. Z.; Wang, W. X. Development of a Chlorine Chemistry Module for the Master Chemical Mechanism. *Geosci Model Dev* **2015**, *8* (10), 3151–3162. https://doi.org/10.5194/gmd-8-3151-2015.

(5) Bertram, T. H.; Thornton, J. A.; Riedel, T. P.; Middlebrook, A. M.; Bahreini, R.; Bates, T. S.; Quinn, P. K.; Coffman, D. J. Direct Observations of N2O5 Reactivity on Ambient Aerosol Particles. *Geophys Res Lett* **2009**, *36*, L19803, doi: 10.1029/2009GL040248.

(6) Bertram, T. H.; Thornton, J. A. Toward a General Parameterization of N2O5 Reactivity on Aqueous Particles: The Competing Effects of Particle Liquid Water, Nitrate and Chloride. *Atmos Chem Phys* **2009**, *9* (21), 8351–8363.

(7) Thornton, J. A.; Kercher, J. P.; Riedel, T. P.; Wagner, N. L.; Cozic, J.; Holloway, J. S.; Dube, W. P.; Wolfe, G. M.; Quinn, P. K.; Middlebrook, A. M.; Alexander, B.; Brown, S. S. A Large Atomic Chlorine Source Inferred from Mid-Continental Reactive Nitrogen Chemistry. *Nature* **2010**, *464* (11), 271–274. https://doi.org/10.1038/nature08905.

(8) Mielke, L. H.; Stutz, J.; Tsai, C.; Hurlock, S.; Roberts, J. M.; Veres, P. R.; Froyd, K.; Hayes, P.; Cubison, M.; Jimenez, J. L.; Washenfelder, R. A.; Young, C. J.; Gilman, J. B.; de Gouw, J.; Flynn, J.; Grossberg, N.; Lefer, B.; Liu, J.; Weber, R.; Osthoff, H. D. Heterogeneous Formation of Nitryl Chloride and Its Role as a Nocturnal NOx Reservoir Species during CalNex-LA 2010. *Journal of Geophysical Research-Atmospheres* **2013**, *118*, 10638–10652. https://doi.org/10.1002/jgrd.50783.

(9) Young, C. J.; Washenfelder, R. A.; Edwards, P. M.; Parrish, D. D.; Gilman, J. B.; Kuster, W. C.; Mielke, L. H.; Osthoff, H. D.; Tsai, C.; Pikelnaya, O.; Stutz, J.; Veres, P. R.; Roberts, J. M.; Griffith, S.; Dusanter, S.; Stevens, P. S.; Flynn, J.; Grossberg, N.; Lefer, B.; Holloway, J. S.; Peischl, J.; Ryerson, T. B.; Atlas, E. L.; Blake, D. R.; Brown, S. S. Chlorine as a Primary Radical: Evaluation of Methods to Understand Its Role in Initiation of Oxidative Cycles. *Atmos Chem Phys* **2014**, *14*, 3427–3440. https://doi.org/10.5194/acp-14-3427-2014.
